# High prevalence of autoimmune disease in the rare inflammatory bone disorder sternocostoclavicular hyperostosis: survey of a Dutch cohort

**DOI:** 10.1186/s13023-017-0573-9

**Published:** 2017-01-25

**Authors:** Pieter A. Valkema, Clare H. Luymes, Janneke E. Witteveen, Saskia le Cessie, Natasha M. Appelman-Dijkstra, Pancras C. W. Hogendoorn, Neveen A. T. Hamdy

**Affiliations:** 1 Department of Medicine, Division of Endocrinology & Centre for Bone Quality, Leiden University Medical Center, Albinusdreef 2, Leiden, 2333ZA The Netherlands; 2 Department of Public Health and Primary Care, Leiden University Medical Center, Leiden, The Netherlands; 3 Department of Medical Statistics and Bioinformatics, Leiden University Medical Center, Leiden, The Netherlands; 4 Department of Clinical Epidemiology, Leiden University Medical Center, Leiden, The Netherlands; 5 Department of Pathology, Leiden University Medical Center, Leiden, The Netherlands

**Keywords:** Sternocostoclavicular hyperostosis, Palmoplantar pustulosis, Autoimmune disease, Prevalence, Survey, Inflammatory disease, SAPHO syndrome

## Abstract

**Background:**

Sternocostoclavicular hyperostosis (SCCH; ORPHA178311) is a rare inflammatory disorder of the axial skeleton, the precise pathophysiology of which remains to be established. We addressed the potential association of SCCH with autoimmune processes by evaluating the lifetime prevalence of autoimmune disease in 70 patients with adult-onset SCCH and 518 SCCH-unaffected first-degree relatives (parents, siblings and children). Danish hospital registry data for autoimmune diseases were used as reference data.

**Results:**

The mean age of interviewed patients was 56.3 years (range 26–80 years) and 86% were female. Interviewed patients belonged to 63 families, with four families having clusters of 2–3 patients. A diagnosis of at least one autoimmune disease was reported in 20 SCCH patients (29%) and in 47 relatives (9.1%), compared to an estimated 3.9% prevalence of autoimmune disease in the Danish reference population. A diversity of autoimmune diseases was reported in SCCH patients and relatives, most frequently psoriasis vulgaris (14%). Palmoplantar pustulosis was reported by 28 patients (40%). In SCCH patients, inclusion of palmoplantar pustulosis as putative autoimmune disease increased the overall prevalence to 54%.

**Conclusions:**

The high prevalence of autoimmune disease in patients with sternocostoclavicular hyperostosis and their first-degree relatives suggests that autoimmunity may play a role in the still elusive pathophysiology of the intriguing osteogenic response to inflammation observed in this rare bone disorder.

## Background

Sternocostoclavicular hyperostosis (SCCH; ORPHA178311) is a rare inflammatory disorder of the axial skeleton, characterized by chronic osteitis associated with a predominantly osteogenic response. The disorder preferentially affects the sternum, medial end of the clavicles and upper ribs, and is often associated with a characteristic dermatosis: palmoplantar pustulosis (PPP), a chronic, sterile inflammation of the skin of palms and soles [1–3].

**Fig. 1:**
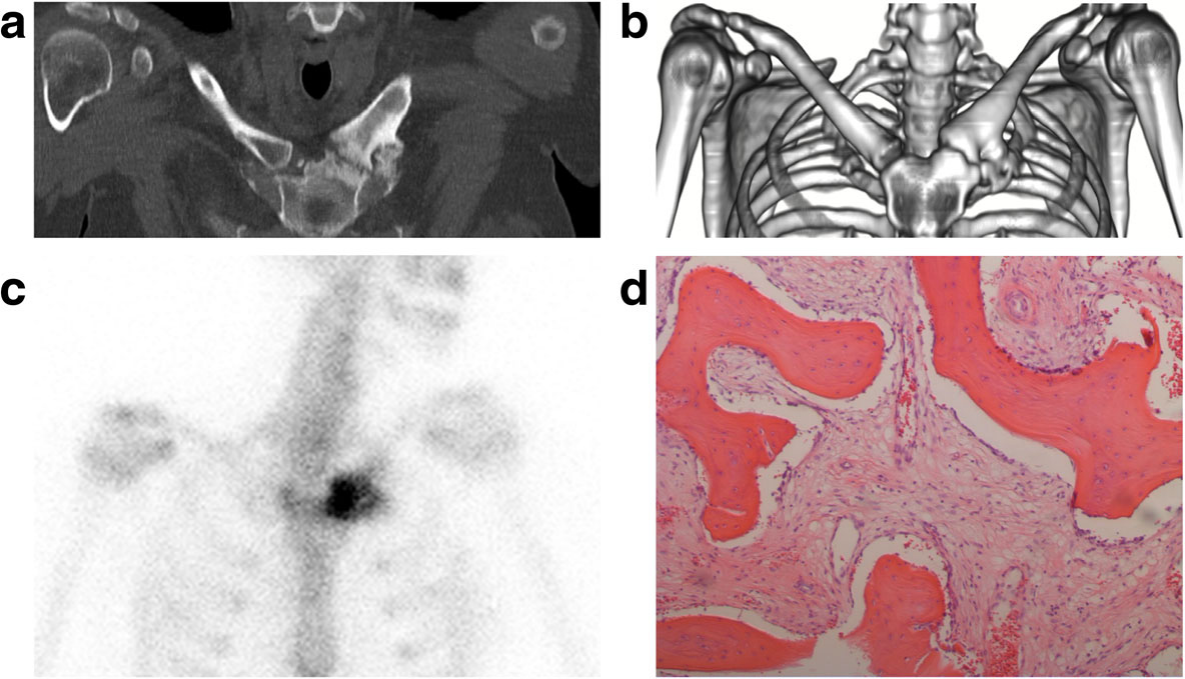
Characteristic radiological, scintigraphic and histological features of sternocostoclavicular hyperostosis in a 33-year-old woman with a 12-year history of SCCH. **a** Computed tomographic (CT) oblique sternal-coronal view of the sternocostoclavicular region showing the characteristic sclerosis and hyperostosis of the medial end of the left clavicle with reactive joint changes corresponding to the site of increased radioisotope uptake on skeletal scintigraphy. **b** Volume-rendered CT-scan showing the characteristic widening of the affected medial end of the left clavicle due to hyperostosis. **c** Technetium-99 m skeletal scintigraphy showing intense uptake of the radioisotope in the left sternoclavicular region. **d** Light micrograph of a biopsy of the bony lesion showing bony trabeculae with irregular kit-lines, with scattered lymphocytes and fibrosis of the marrow (hematoxylin and eosin stain; front objective 10× magnification)

Clinical features of SCCH are pain and swelling of the affected bones, with periods of exacerbation and remission. The diagnosis is established on the basis of characteristic scintigraphic and radiological features (Fig. 1a–c). These consist of focal increases in radioisotope uptake in affected bones on a technetium-99 m bone scintigraphy (Fig. 1c) and of pathognomonic hyperostosis and sclerosis in these bones on computed tomography (CT) (Fig. 1a–b). Histological features are those of a chronic osteomyelitis with inflammation and fibrosis of the marrow cavity, in the absence of an apparent pathogen (Fig. 1d) [2–4].

The inflammatory osteitis of SCCH is part of the SAPHO syndrome (*Synovitis, Acne, Pustulosis, Hyperostosis and Osteitis*; ORPHA793), which describes the variable association of the skeletal manifestations of SCCH with inflammatory joint manifestations, such as peripheral synovitis, and inflammatory cutaneous manifestations, such as palmoplantar pustulosis, severe acne, hidradenitis suppurativa and pustular psoriasis [3–9]. SCCH is also part of the skeletal manifestations of chronic recurrent multifocal osteomyelitis of children (CRMO; ORPHA324964). Notwithstanding, the isolated skeletal manifestation of SCCH (ORPHA178311) is also recognized as a separate entity [1].

The precise pathophysiology of the chronic osteitis observed in these syndromes remains elusive. There is, however, increasing evidence for a contributory role of inflammatory, autoimmune and genetic factors in the pathophysiology of the skeletal, articular and cutaneous manifestations of these multi-faceted syndromes. Family clustering has been reported for the SAPHO syndrome [10–12] and for CRMO in children [13, 14]. We have observed familial clustering for isolated adult-onset SCCH (unpublished observations). There have also been a number of reports of family clustering of a variety of autoimmune diseases in SAPHO and CRMO families [11–15]. Autoimmunity and autoinflammation have been shown to be closely related and not to be mutually exclusive [16]. There is also evidence for a strong heritable component of autoimmune diseases in general, with family clustering being a commonly observed phenomenon [17, 18].

The potential role of autoimmunity in the pathophysiology of the osteoarticular and cutaneous manifestations of SCCH and SAPHO has not been fully explored. However, an association between chronic inflammatory osteitis and psoriasis, a chronic, immune-mediated inflammatory skin disease has been consistently observed [6, 11, 19–21]. Moreover, palmoplantar pustulosis (which is frequently present in SCCH patients) has been proposed to be an autoimmune disease in its own right [22, 23].

To our knowledge, there has been so far no systematic evaluation of general autoimmune comorbidity or of increased prevalence of autoimmune disease in relatives of patients with adult-onset SCCH. The objective of this study was to determine the lifetime prevalence of autoimmune disease in patients with adult-onset SCCH and their first-degree relatives by means of a structured telephone survey of index patients, during which information was also collected on the prevalence of these disorders in first-degree relatives. This approach may provide new insights into the potential role of autoimmunity and reinforce the evidence for genetic susceptibility in the pathogenesis of the disorder.

## Methods

We conducted a cross-sectional structured telephone survey in patients with a diagnosis of adult-onset SCCH, established on the basis of characteristic clinical, scintigraphic and radiological features. The majority of patients (81%) were identified from the Leiden University Medical Center’s hospital registry. A minority of patients (19%), not followed up in our out-patient clinic, but in whom the diagnosis of SCCH was also confirmed, were further recruited from the membership of the Dutch SCCH Patients’ Association (http://www.scch.nl).

All patients were contacted by telephone and invited to take part in the survey. An appointment for the actual telephone interview was made during this call, after verbally obtaining the patient’s informed consent to take part in the survey and to allow the use of additional clinical information from their medical records. For patients not followed up in our Center, written consent was additionally obtained to request relevant medical information from their treating physician.

The study was approved by the Medical Ethical Committee of the Leiden University Medical Center (Protocol registration number P08.112).

The interview was planned for a duration of 30–45 min and structured to include questions relating to demographic details, relevant medical history including past and present clinical manifestations of SCCH (pain, swelling, local inflammatory changes, other), date of onset of symptoms, date of diagnosis, investigations performed and treatment received. A family pedigree, bridging three generations was constructed with information on present and past history of autoimmune disease, collected for the patient as well as for affected or unaffected biological relatives in the first degree: parents, full siblings and children. The precise age of unaffected first-degree relatives was not recorded.

Self-reported diagnoses of autoimmune diseases were verified with information obtained from the patients’ medical records or from that obtained from the patients’ treating physicians in case the patient had never been seen in our institution. Diagnoses reported by patients for first-degree relatives were not cross-checked. Pathognomonic features of palmoplantar pustulosis, a chronic skin condition in which tiny sterile fluid/pus filled pustules appear in waves of variable severity on one or both palms and/or soles, were considered to be sufficiently specific to warrant a diagnosis of the condition without further confirmation. We considered a reported diagnosis for a patient or his or her first-degree relatives to represent an autoimmune disease when the diagnosis corresponded to one of the 30 autoimmune diseases listed by Eaton et al. in their 2010 publication on the prevalence of 30 ICD-8 and ICD-10-classified autoimmune diseases in Denmark [24]. Diagnoses reported in first-degree relatives were discarded when too vague to be reliably identified with a diagnosis on Eaton’s list (for instance, we considered a reported diagnosis of ‘hypothyroidism’ without further specification as insufficient to be recorded as ‘autoimmune thyroiditis’). The diagnosis “mixed connective tissue disease”, representing a specific syndrome of a number of concomitant autoimmune disorders, was added to Eaton’s list and studied as a separate category.

To compare the prevalence of autoimmune diseases in SCCH patients and their first-degree relatives to that of the general population, we used the above-named epidemiologic data from combined ICD-8 and ICD-10 hospital registrations in Denmark [24]. These data include lifetime prevalence estimates for 30 autoimmune diseases, as well as an aggregate prevalence estimate which corrects for individuals having a history of more than one autoimmune disease [24]. Similar data on the global epidemiology of autoimmune diseases are not available for the general Dutch population. However, the Netherlands and Denmark are culturally, ethnically and socio-economically comparable, and the countries have similar healthcare systems [25].

### Statistical analysis

The primary outcome of the study was the overall lifetime prevalence of autoimmune disease in index patients and their first-degree relatives. Prevalence was compared between groups using the chi-square test. Mean values between groups were compared using the unpaired *t*-test. Comparisons with the general Danish population were made using the one sample z-test. A significance threshold of *p* < 0.05 was used. SPSS Statistics version 20 (Armonk, NY, United States: IBM Corp.) was used for all analyses.

## Results

**Fig. 2:**
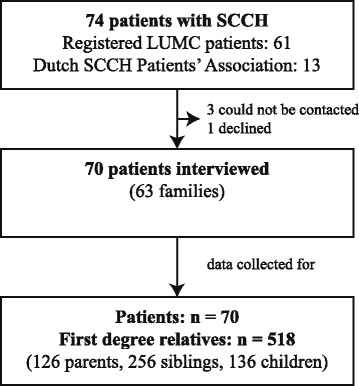
Flowchart of the patients included in the study

Of 74 known patients with an established diagnosis of adult-onset SCCH, three could not be contacted and one declined to take part in the survey, so that a total of 70 patients were finally interviewed (Fig. 2). The 70 interviewed index patients reported data on 518 first-degree family relatives unaffected by SCCH. The patients belonged to 63 separate families, four of which had multiple affected family members. Three families had three confirmed patients at the time of enquiry (mother, son and daughter; brother and two sisters; mother and two daughters), and one family had two confirmed patients (mother and daughter).

### Demographic characteristics

SCCH patients were predominantly female (86%) and had a mean age of 56.3 ± 13.6 years at the time of the interview (range 26–80 years). There was no significant difference in age between interviewed men (mean age 53.8 ± 8.6 years) and women (mean age 56.7 ± 14.3 years; *p* = 0.53).

### Prevalence of autoimmune disease in index patients and unaffected first-degree relatives

A history of at least one autoimmune disease was present in 20 of the 70 patients (29%, 95% CI [19–40%]) and in 47 of the 518 first-degree relatives (9.1%, 95% CI [6.9–11.9%]). The lifetime prevalence of autoimmune disease did not significantly differ between men and women, in patients (40% in men, 27% in women, *p* = 0.39) or in their first-degree relatives (7.3% in men, 10.7% in women, *p* = 0.19).

In patients with SCCH, the most frequently encountered autoimmune disease was psoriasis vulgaris (14% of patients), compared to a prevalence of 0.32%, 95% CI [0.32%–0.32%] in the Danish general population [24] (Table 1). In a separate analysis excluding psoriasis from the list of autoimmune diseases, the prevalence of these remained high with 17% of patients and 7.3% of relatives with a history of at least one autoimmune disease other than psoriasis. These figures are still significantly higher than the reference prevalence in the general population of 3.9%, 95% CI [3.92–3.96%], (*p* < 0.001) [24].

**Table 1:** Prevalence of autoimmune diseases in SCCH patients and first-degree relatives

	Patients	Relatives	Relatives per generation			Denmark^a^
			Child	Sibling	Parent	
Total N	70	518	136	256	126	5 506 574
Female sex	60 (86%)	272 (52.5%)	69 (50.7%)	140 (54.7%)	63 (50.0%)	50.5^b^
Age (mean ± SD)	56.3 ± 13.6	ND	ND	ND	ND	39.8^b^
Autoimmune diseases						
Psoriasis vulgaris	10 (14%)	10 (1.9%)	4 (2.9%)	3 (1.2%)	3 (2.4%)	0.32%
Alopecia areata	0	2 (0.4%)	1 (0.7%)	1 (0.4%)	0	0.03%
Rheumatoid arthritis	2 (3%)	15 (2.2%)	1 (0.7%)	5 (2.0%)	9 (7.1%)	0.50%
Ankylosing spondylitis	2 (3%)	1 (0.2%)	0	0	0	0.07%
Polymyalgia rheumatica	1 (1%)	1 (0.2%)	0	0	1 (0.8%)	0.27%
Crohn’s disease	0	3 (0.6%)	1 (0.7%)	1 (0.4%)	1 (0.8%)	0.23%
Ulcerative colitis	0	4 (0.8%)	2 (1.5%)	2 (0.8%)	0	0.48%
Coeliac disease	0	3 (0.6%)	0	2 (0.8%)	1 (0.8%)	0.06%
Thyrotoxicosis	1 (1%)	2 (0.4%)	1 (0.7%)	1 (0.4%)	0	0.45%
Autoimmune thyroiditis	3 (4%)	1 (0.2%)	1 (0.7%)	0	0	0.06%
Diabetes mellitus type 1	1 (1%)	2 (0.4%)	2 (1.5%)	0	0	0.91%
Multiple sclerosis	0	2 (0.4%)	1 (0.7%)	1 (0.4%)	0	0.19%
Pernicious anemia	1 (1%)	3 (0.6%)	0	2 (0.8%)	1 (0.8%)	0.05%
MCD/overlap syndrome^c^	1 (1%)	0	0	0	0	ND
Scleroderma	0	1 (0.2%)	0	0	1 (0.8%)	0.03%
Uveitis anterior	1 (1%)	0	0	0	0	0.21%
PPP *(putative)*	28 (40%)	9 (1.7%)	2 (1.5%)	6 (2.3%)	1 (0.8%)	ND
Any autoimmune disease^d^	20 (29%)	47 (9.1%)	13 (9.6%)	18 (7.0%)	16 (12.7%)	3.9%
Any AID, including PPP^d^	38 (54%)	56 (10.8%)	15 (11.0%)	24 (9.4%)	17 (13.5%)	ND
Any AID, excluding psoriasis^d^	12 (17%)	38 (7.3%)	10 (7.4%)	15 (5.9%)	13 (10.3%)	ND

*Abbreviations*: *SD* standard deviation, *ND* no data, *PPP* palmoplantar pustulosis, *MCD* mixed connective tissue disease, *AID* autoimmune disease

^a^Danish general population prevalence data on 30 autoimmune diseases (from ICD-8 and ICD-10 codes), adapted from Eaton et al. [24]

^b^Demographic characteristics as of 1 January 2006 adapted from StatBank Denmark (http://statbank.dk/). Sex ratio and mean age respectively were retrieved from the FOLK2 and GALDER datasets

^c^This patient was analysed in a separate category; she had concomitant diagnoses of MCD and Sjögren’s syndrome

^d^Reported figures take into account individuals with multiple diagnoses. Four patients and two relatives had two autoimmune diseases

Four SCCH patients reported more than one diagnosis of autoimmune disease. One patient had concurrent diagnoses of psoriasis and rheumatoid arthritis. A second patient had an autoimmune overlap syndrome with concomitant diagnoses of mixed connective tissue disease and Sjögren’s syndrome. A third patient had a history of polymyalgia rheumatica and autoimmune thyroiditis. A fourth patient had ankylosing spondylitis and psoriasis.

Among first-degree relatives, two individuals had a history of more than one autoimmune disease (Crohn’s disease and rheumatoid arthritis in one, and autoimmune thyroiditis and psoriasis in a second).

### Prevalence of associated inflammatory conditions not classified as autoimmune disease

A number of inflammatory conditions not classified as autoimmune disease in Eaton’s listing were reported during the survey. The most commonly reported inflammatory skin condition was the pathognomonic palmoplantar pustulosis in 28 index patients (40%) and nine first-degree relatives (1.9%). Data on the prevalence of this skin condition are not available for the general population. Other inflammatory skin conditions reported included severe acne on the anterior chest or back (six patients and five relatives), pyoderma gangrenosum (one patient), hidradenitis suppurativa (one patient) and Jessner lymphocytic infiltrate (one patient). One patient had a diagnosis of polyarteritis nodosa. Löfgren syndrome (an acute variant of sarcoidosis) was reported in a sibling of one of the patients.

Although none of the patients had a confirmed diagnosis of inflammatory bowel disease, non-specific gastrointestinal symptoms ranging from chronic diarrhea to constipation were reported by 27 (39%) of the patients.

### Effect of age on the prevalence of autoimmune disease

To evaluate whether a history of autoimmune disease was associated with age in SCCH, we stratified patients using the following age categories: below 40 years, between 40 and 60 years and 60 years and older. In the youngest, middle and oldest strata, three out of seven (43%), 10 out of 33 (30%) and seven out of 33 (23%) patients respectively had a history of autoimmune disease. There was no significant difference between age groups (*p* = 0.56).

To assess the effect of age on the prevalence of autoimmune disease in first-degree relatives, we subdivided individuals in this group into three generations, siblings of patients (*n* = 256), parents (*n* = 126) and children (*n* = 136). In the parent, sibling and children generations, 13 (9.6%), 18 (7.0%) and 16 (12.7%) individuals respectively had a history of autoimmune disease (Table 1). There was no significant difference in prevalence of autoimmune disease between generations in unaffected first-degree relatives (*p* = 0.19).

## Discussion

Our findings from this study demonstrate a relatively high life-time prevalence of autoimmune disease in 29% of patients with adult-onset SCCH and in 9% of their first-degree relatives, compared to prevalences varying from 3.2%, based on a literature review of studies published between 1965 and 1995 on 24 autoimmune diseases [13], to 3.9% based on national hospital registry data for 30 diseases in Denmark [24]. The observed higher prevalence of autoimmune disease is also higher than the prevalence of 7.6–9.4% as calculated after applying a correction to Eaton et al.’s estimates for six diseases for which reliance on hospital data may have produced an underestimation of true prevalence (alopecia, celiac disease, hyperthyroidism, hypothyroidism, psoriasis and vitiligo) [26].

Our data support a tendency for autoimmune diseases to occur at greater than expected rates in patients with SCCH and their first-degree relatives. Although the increase in the prevalence of autoimmune disease in our cohort of patients was general, it was also heterogeneous in nature, not appearing to be a uniform phenomenon across all diseases (except for psoriasis, which was particularly prevalent, disproportionately contributing to the excess prevalence of autoimmune disease in index patients as well as in their first-degree relatives).

It is thought that autoimmune disease develops in genetically susceptible individuals, with expression of the disease modified by environmental factors [17]. Our finding of clustering of autoimmune diseases in SCCH patients and their first-degree relatives, although suggesting the contribution of a general genetic background of autoimmunity to the pathogenesis of this complex disorder, does not necessarily imply a direct cause-effect relationship between autoimmune diseases and SCCH. Ultimately, other underlying factors may well be responsible for both, although identifying such factors may prove to be difficult.

The abnormal osteogenic response to inflammation characteristic of SCCH is interestingly reminiscent of that observed in the seronegative spondyloartropathy ankylosing spondylitis, with a number of studies currently investigating the inflammatory and immune underpinnings of this disorder [27, 28].

An interesting hypothetical framework that may explain the association between inflammation and autoimmunity can be found in the proposed immunological “danger theory”, which emphasizes that autoimmune disease can be provoked by cellular damage (“danger”) signals [29, 30]. Within this context, it could be speculated that in SCCH patients, prolonged exposure to low-grade inflammation combined with an inadequate clearance of self-antigens from cellular debris may be associated with a state of increased susceptibility to autoimmune disease in general.

Psoriasis is a chronic, immune-mediated inflammatory skin disease estimated to affect about 2–4% of the population in western countries. Our data on the high prevalence of psoriasis in adult-onset SCCH are in keeping with those reported in patients with the SAPHO syndrome [11] and with data in children with CRMO [13].

The high prevalence of a specific autoimmune disease such as psoriasis in affected as well as unaffected members of SCCH families, in excess to that expected by chance, may additionally offer insight into shared pathophysiologic mechanisms that may lead to unraveling shared etiological pathways.

Palmoplantar pustulosis was also frequently reported, although its classification as an autoimmune disease is as yet not widely accepted. Palmoplantar pustulosis was originally believed to be a variant of psoriasis vulgaris (to which it is closely related), although the condition has distinct characteristics setting it apart from psoriasis [31, 32]. The frequent occurrence of both psoriasis and palmoplantar pustulosis suggests that these diseases may be closely linked to the pathogenesis of SCCH. Further understanding of the disease pathways involved in the pathogenesis of these disorders will undoubtedly help to delineate how exactly they may be interrelated.

An association has been previously reported between inflammatory bowel disease (IBD) and the SAPHO syndrome [13, 15, 33]. Intriguingly, none of our index SCCH patients had a confirmed diagnosis of IBD, although non-specific gastrointestinal symptoms were frequently reported. Whereas, in our population, underdiagnosis of IBD cannot be excluded, intestinal manifestations in SCCH patients, albeit non-specific, may also represent an independent inflammatory phenomenon outside the context of IBD. Recent insights from the *Pstpip2*-deficient mouse model suggest that this is plausible [34, 35]. In these osteomyelitis-susceptible mice, alterations in the intestinal microbiota were thus found to accompany manifestations of inflammatory bone disease. Interestingly, a change in dietary composition prevented both the microbiota changes and the inflammatory bone changes.

Our study has strengths as well as limitations. To our knowledge, this is the first study addressing the prevalence of autoimmune disease in a cohort of patients with adult-onset SCCH and their first-degree relatives. First-hand information was available for all patients, the vast majority of whom were diagnosed and followed up in our Out-patient Clinic for a number of years. This allowed us to verify the self-reported diagnoses of autoimmune disease obtained during the telephone interview with the patients’ medical records, providing some degree of confidence on the accuracy and completeness of the reported medical histories.

Our study has also a number of limitations. The data obtained from SCCH patients about their unaffected first-degree relatives are arguably less reliable, as not verifiable. Unaffected family members were not separately contacted, and data provided by patients about their relatives were not cross-checked with their medical records. Notwithstanding, the process of systematic family histories has been successfully used in the past to investigate familial clustering of autoimmune diseases [18].

We recognize that observational bias is a common problem with retrospective surveys, and that such a bias may have influenced our estimates. Misclassification may have occurred due to misdiagnosis or underdiagnosis of an autoimmune disease. It is also possible that some of the reported diagnoses may have been based on criteria established some years ago, which may be no longer valid in the modern era. For example, a diagnosis of rheumatoid arthritis established decades ago may need to be reappraised. It is also possible that the prevalence of SCCH itself could have been underestimated in our families–for instance, family members with locomotor symptoms may have undiagnosed SCCH. We have actually previously reported on the frequent delay in diagnosing SCCH, which could last for several years [36].

It has been argued that the figure of 3.9%, which we have used in our study as reference value for the prevalence of autoimmune disease in the general population [24], may be an underestimate of the true prevalence of autoimmune disease, as it is based on Danish hospital registrations [26]. Nonetheless, the high lifetime prevalence of autoimmune disease of 29% observed in SCCH patients is sufficiently high to preclude it being a chance finding.

Another potential limitation in the interpretation of our data is that our surveyed cohort of adult-onset SCCH patients is relatively older than that of the reported Danish general population (Table 1). However, we found no significant association between age and the prevalence of autoimmune disease in SCCH patients, nor did we find significant inter-generation differences in the prevalence of autoimmune diseases in first-degree relatives of index patients.

## Conclusions

Our data from this study demonstrate a high overall prevalence of autoimmune disease in patients with adult-onset SCCH and their unaffected first-degree relatives and provide further evidence for the specific, strong association of SCCH with palmoplantar pustulosis and psoriasis vulgaris. Whereas the association between SCCH and autoimmune disorders shown in our study is no proof of causality, this finding does raise the hypothesis of a potential role of a common pathway for autoimmunity in the still elusive pathophysiology of the intriguing abnormal osteogenic response to inflammation observed in this rare bone disorder.
